# Conserved NCoR1 decline underlies small intestinal aging

**DOI:** 10.1093/lifemedi/lnag035

**Published:** 2026-09-22

**Authors:** Yunuo Mao, Huili Hu

**Affiliations:** The Key Laboratory of Experimental Teratology, Ministry of Education, Department of Systems Biomedicine, School of Basic Medical Sciences, Shandong University, Jinan 250012, China; The Key Laboratory of Experimental Teratology, Ministry of Education, Department of Systems Biomedicine, School of Basic Medical Sciences, Shandong University, Jinan 250012, China; Department of Geriatric Medicine & Laboratory of Gerontology and Anti-Aging Research, Qilu Hospital, Cheeloo College of Medicine, Shandong University, Jinan 250012, China

Defining the cellular and molecular basis of intestinal aging remains an important question in aging biology. The small intestine is a highly organized, continuously renewing tissue, in which stem cells and differentiated epithelial lineages are coordinated within a tightly patterned crypt–villus unit. This architecture sustains nutrient uptake, epithelial repair, and barrier protection over the lifespan [1]. During aging, however, these functions progressively decline. The small intestinal epithelium loses integrity and resilience, increasing susceptibility to bacterial overgrowth, systemic metabolic disturbance, and immune dysregulation [2]. Prior work implicated defects in both stem cells and their niche, including altered Paneth-cell signaling with the loss of regenerative capacity [3]. Recent single-cell studies further suggest that aging reshapes intestinal stem-cell states, promotes inflammatory signaling, and biases epithelial differentiation toward secretory programs [4]. However, the regulatory factors that coordinate these age-associated epithelial changes and their responsiveness to pharmacological anti-aging interventions remain incompletely defined.

In a recent issue of *Nature Aging*, Li et al. [5] address this question by generating a single-nucleus atlas of small intestinal aging in cynomolgus monkeys. Through combining single-nucleus transcriptomics, organoid perturbations, and long-term metformin treatment, the study provides mechanistic insight into primate intestinal aging and identifies nuclear receptor corepressor 1 (NCoR1) as a pharmacologically responsive regulator of age-associated epithelial decline. These results highlight NCoR1 as a potential molecular node for understanding and potentially modulating age-associated intestinal dysfunction.

Li et al. first characterized the aged primate intestine at the tissue level. Aged cynomolgus monkeys displayed shortened villi and crypts, increased senescence-associated β-galactosidase activity, and elevated oxidative stress and apoptosis. Epithelial proliferation was reduced, whereas junctional organization was impaired, as reflected by decreased levels of zonula occludens-1 (ZO-1) and E-cadherin. The authors then generated a single-nucleus transcriptomic atlas resolving major epithelial and non-epithelial compartments and reconstructed the epithelial continuum from intestinal stem cells through absorptive and secretory lineages. Aging increased transcriptional noise, reduced cell identity scores, and activated inflammatory and stress programs across epithelial populations, indicating a broad loss of transcriptional fidelity. The intestinal stem-cell compartment was particularly affected, with reduced Wnt, Notch, and fatty-acid oxidation programs, together with increased stress and mechanistic target of rapamycin complex 1 (mTORC1) signaling. Trajectory analysis further suggested that aging altered differentiation along the crypt–villus axis and weakened absorptive programs, while shifting epithelial output toward secretory and Paneth-cell states (Fig. 1A). Collectively, these findings suggest that primate intestinal aging involves not only reduced proliferation, but also disrupted lineage fidelity and biased epithelial differentiation.

**Figure 1.**
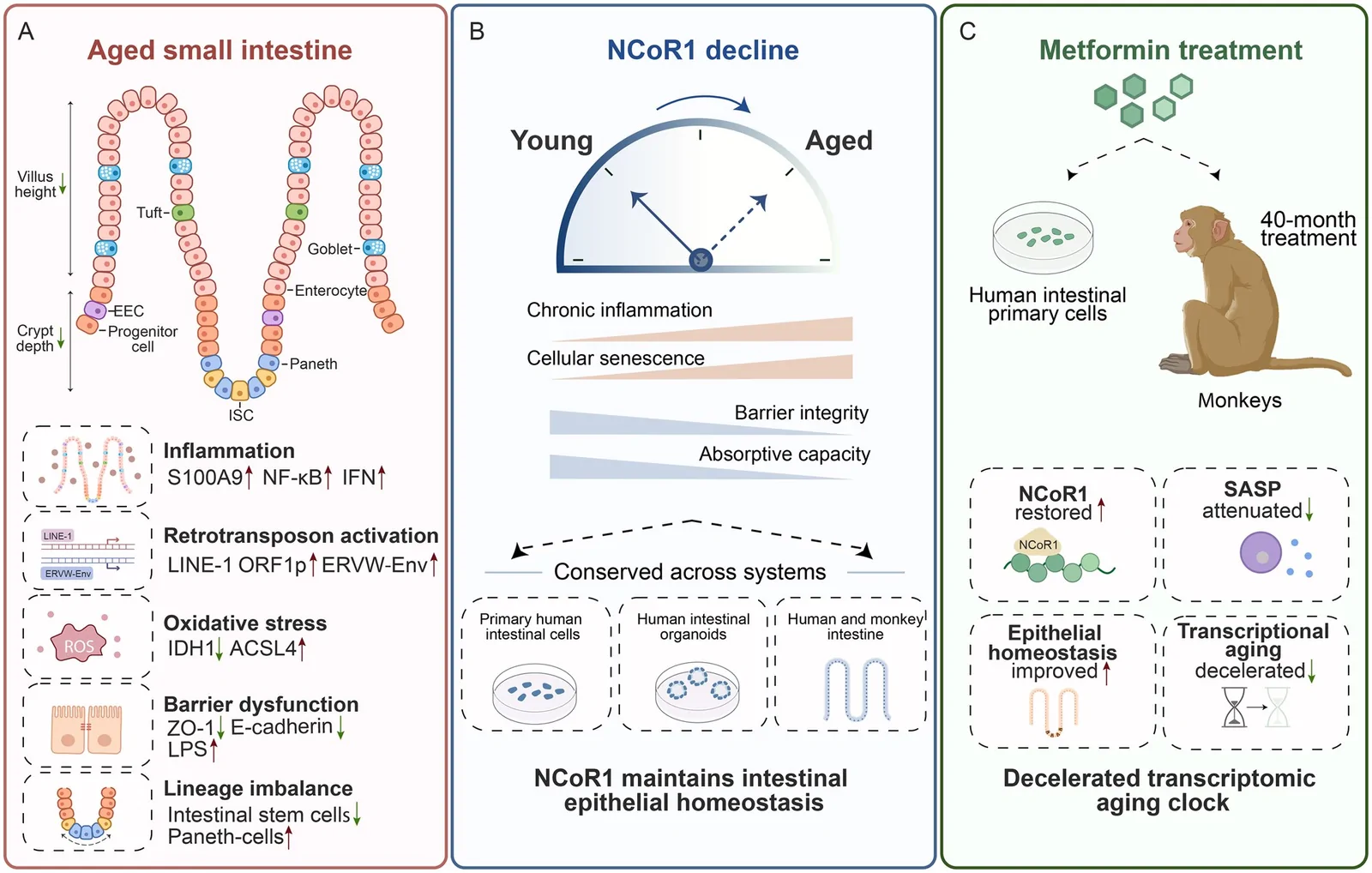
Single-nucleus profiling reveals metformin-reversible NCoR1 decline in small intestinal aging. (A) Schematic summary of age-associated changes in the small intestine. (B) NCoR1 declines during intestinal aging and functions as a conserved regulator of epithelial homeostasis. (C) Metformin restores NCoR1-associated epithelial homeostasis. Abbreviations: ACSL4, acyl-CoA synthetase long-chain family member 4; EEC, enteroendocrine cells; ERVW-Env, endogenous retrovirus group W envelope protein; IDH1, isocitrate dehydrogenase 1; IFN, interferon; ISC, intestinal stem cells; LINE-1, long interspersed nuclear element-1; LPS, lipopolysaccharide; NCoR1, nuclear receptor corepressor 1; NF-κB, nuclear factor κB; ORF1p, open reading frame 1 protein; SASP, senescence-associated secretory phenotype; S100A9, S100 calcium-binding protein A9; ZO-1, zonula occludens-1.

To identify candidate regulators of age-associated epithelial changes, the authors integrated differential expression, aging-gene annotation, and transcriptional regulatory analyses. NCoR1 emerged as a broadly downregulated transcriptional corepressor in the aged intestine—a pattern also seen in human intestinal samples. Reduced NCoR1 correlated with features of the aged epithelium, including diminished proliferation, inflammatory activation, and Paneth-cell expansion. Functional perturbation in human epithelial cells and intestinal organoids supported a causal role for NCoR1 loss. In primary small intestinal epithelial cells, *NCoR1* knockdown induced senescence, DNA damage, and proliferative arrest. Organoid experiments extended these observations in a 3D epithelial system that retains self-renewal and multilineage differentiation. Conversely, CRISPR activation (CRISPRa)-mediated activation of *NCoR1* attenuated oxidative-stress-induced senescence in epithelial cells, reducing senescence-associated β-galactosidase (SA-β-gal) activity, p21 expression, and DNA damage while restoring proliferation and heterochromatin organization (Fig. 1B). Together, these loss- and gain-of-function experiments indicate that NCoR1 is not merely a marker of aging, but an active regulator whose decline recapitulates key features of epithelial aging.

The authors next probed whether the NCoR1-regulated program is pharmacologically modifiable. They focused on metformin, a widely used clinical drug with reported anti-aging effects. In human primary small intestinal epithelial cells, metformin increased NCoR1 protein levels and reduced senescence-associated phenotypes. These protective effects were attenuated by NCoR1 depletion, suggesting that NCoR1 contributes to the epithelial response to metformin. In aged cynomolgus monkeys treated with metformin over the long term, intestinal NCoR1 expression was restored, inflammatory activity was reduced, and epithelial proliferation was improved. A single-cell transcriptomic aging clock further suggested that metformin slowed the molecular aging trajectory of the intestine (Fig. 1C). These results indicate that NCoR1-associated transcriptional programs remain responsive to pharmacological modulation in the aged primate intestine, further reinforcing NCoR1’s role in age-associated epithelial decline.

Li et al. validated NCoR1’s involvement in intestinal aging through cross-species validation and human-derived experimental models. NCoR1 decline was observed across multiple cell types in aged cynomolgus monkey intestine and also detected in human intestinal tissues, indicating that this change was conserved. Perturbation in primary human epithelial cells showed that NCoR1 loss induced senescence, DNA damage, and junctional disruption, while organoids provided a tractable system to assess effects on epithelial growth, stem-cell activity, and lineage differentiation. These complementary approaches strengthen the conclusion that NCoR1 participates in the regulation of intestinal aging.

Moreover, metformin has been reported in multiple studies to attenuate aging-associated phenotypes [6], and recent long-term work in cynomolgus monkeys further showed that this clinically used drug can slow aging-clock progression across tissues [7]. In the intestine, metformin has been reported to attenuate stem-cell dysfunction, barrier impairment, and inflammation in invertebrate and rodent models, supporting the beneficial effects of metformin on intestinal homeostasis [8, 9]. Li et al. extend these observations by linking metformin responsiveness to NCoR1, a conserved epithelial regulator identified at single-cell resolution. Rather than merely confirming the broad metabolic and anti-inflammatory effects of metformin, the study provides a mechanistic framework in which restoration of NCoR1 is associated with improved epithelial homeostasis and decelerated intestinal molecular aging. The recovery of NCoR1 in metformin-treated aged primates provides *in vivo* support for its role in intestinal aging.

This work also raises important questions. The upstream drivers of NCoR1 decline during aging are not yet defined. Chronic inflammation, oxidative stress, microbial metabolites, and epigenetic remodeling could each contribute. Although NCoR1 appears to mediate part of the epithelial response to metformin, whether it is a direct pharmacological target or a downstream mediator of broader metabolic remodeling remains unresolved. Cell-type-specific manipulation of NCoR1 *in vivo*—particularly in aged primates—was not feasible here and will be important to establish causal roles in distinct compartments. Given metformin’s pleiotropic effects, dissecting the extent to which NCoR1 directly mediates its intestinal benefits remains necessary. Finally, it will also be important to examine whether NCoR1 decline contributes to age-associated intestinal disorders beyond physiological aging, including inflammatory bowel disease and impaired repair after injury. Addressing these questions will clarify how NCoR1-centered regulation integrates local epithelial programs with systemic anti-aging interventions and whether it represents a broader determinant of intestinal epithelial resilience.

Overall, Li et al. provide a valuable resource and mechanistic framework for understanding primate small intestinal aging, identifying NCoR1 as a conserved regulator of age-associated epithelial decline. By showing that metformin restores NCoR1 expression and slows intestinal transcriptomic aging in aged primates, the study suggests that this regulatory program remains pharmacologically responsive. These findings highlight NCoR1-associated transcriptional homeostasis as a promising strategy for preserving intestinal function during aging.
